# Evaluation of a peer counselling programme to sustain breastfeeding practice in Hong Kong

**DOI:** 10.1186/1746-4358-2-12

**Published:** 2007-09-20

**Authors:** Esther HY Wong, EAS Nelson, Kai-Chow Choi, Kin-Ping Wong, Carmen Ip, Lau-Cheung Ho

**Affiliations:** 1 Department of Paediatrics, The Chinese University of Hong Kong, Hong Kong; 2 Centre for Epidemiology and Biostatistics, The Chinese University of Hong Kong, Hong Kong; 3 Princess Margaret Hospital, Hong Kong

## Abstract

**Background:**

Peer counselling is reported to increase breastfeeding rates. We evaluated an intervention consisting of mainly telephone contact peer counselling programme on breastfeeding duration and exclusivity.

**Methods:**

Peer counsellors (PCs) were mothers who had successfully breastfed and had received formal training. Following a postnatal visit, they provided scheduled telephone consultations (Days 1, 4, 7, Weeks 2, 4, 8, and Month 4) to PC group mothers (n = 100) who continued breastfeeding their infants after discharge. Control group mothers (n = 100) received routine care.

**Results:**

After adjusting for mothers' previous breastfeeding experiences, mothers' working status and breastfeeding problems, no statistical differences in mothers' feeding methods (exclusive, almost exclusive or predominant breastfeeding) were noted at the three follow-up times for intervention and control mothers respectively (Day 5: 37%/38%, 46%/53%, 57%/63%; Month 3: 10%/9%, 17%/23%, 20%/26%; Month 6: 2%/1%, 18%/18%, 18%/19%). All differences between the groups were not significant. Also, there was no evidence to suggest that PC intervention prolonged breastfeeding duration.

**Conclusion:**

The lack of effect of our PC intervention may reflect the low baseline breastfeeding rate and low value placed on breastfeeding in our population, the type of PC intervention or group allocation biases.

**Trial registration:**

ISRCTN93605280.

## Background

At the fifty-fifth World Health Assembly, the World Health Organization (WHO) recommended that optimal infant nutrition was exclusive breastfeeding for the first six months of life, followed by the introduction of nutritionally adequate and safe complementary feeding with continued breastfeeding for up to the age of two years or beyond [1]. Despite the many advantages of breastfeeding, the majority of Hong Kong mothers do not attain this goal. Hong Kong's breastfeeding initiation rate, exclusive breastfeeding rate and overall breastfeeding duration are low compared to other countries [2]. Over a ten year period (1987 to 1997), rates of breastfeeding for more than three months increased from 3.9% to 10.3% [2]. Based on data from maternity units, the percentage of newborns ever breastfed in Hong Kong rose from a level of 10% in 1981 to around 20% for the period 1982 to 1992, and then steadily rose to reach 60% in 2001 [3]. The Department of Health has conducted annual breastfeeding surveys since 1998 at Maternal and Child Health centres (MCHC) which have shown breastfeeding beyond one year has increased from 2% (1997) to 5% (2000) [3]. Despite these improving trends, Hong Kong has a high rate of infant formula feeding compared with other countries. During 1996/1997, an international comparative study of child care practices at three months of age, involving 21 centres in 17 countries, showed that Hong Kong had the lowest rate of breastfeeding only (4%) and the highest rate of infant formula feeding only (87%) of all participating centres [4].

A number of strategies can significantly improve and sustain breastfeeding practice, including community-based breastfeeding peer counsellor (PC) programmes (mother-to-mother support) [5-8]. These peer support programmes, either home visit or telephone consultation, have generally been shown to improve breastfeeding initiation, duration and exclusivity.

## Methods

### Participants

The study was undertaken at one government hospital in Hong Kong that had active breastfeeding support and had recently started a PC volunteer programme. Mothers were eligible for inclusion in the study if they were Cantonese-speakers, healthy and had had a vaginal delivery of a full term healthy infant. It was required that mothers planned to stay in Hong Kong for six months postpartum, and that they expressed an intention to breastfeed upon admission to the postnatal unit.

### Intervention

As part of the hospital's PC volunteer programme, mothers were visited by a PC postpartum on an ad hoc basis. The PC would provide mothers with information on the benefits of exclusive breastfeeding, breastfeeding during illness, basic lactation anatomy and physiology, positioning and "latching-on", common myths, problems and solutions, healthy breastfeeding patterns, maternal concerns, milk expression and storage, and sources of social and community support. If a mother was subsequently recruited into the PC intervention group, she would in addition receive seven regular telephone consultations from a PC (at 24 hours, four days, one week, two weeks, one month, two months and four months post discharge). These contacts were discontinued at any time if the mother decided to completely stop breastfeeding her infant. The PCs were allowed to provide more frequent telephone support if necessary. The control group mothers would not receive a PC visit or any phone contacts. They would receive the usual postnatal care and breastfeeding advice which included antenatal education.

### Objectives

This study was designed to assess whether a programme involving a postpartum PC hospital visit followed by PC telephone support after discharge could be an effective strategy to promote breastfeeding in Hong Kong by increasing the duration and exclusivity of breastfeeding.

### Sample size

A total of 200 mothers were recruited into a PC intervention group (n = 100) and control group (n = 100). The hospital had information on the rate of any breastfeeding rate at discharge, and at one week and one month after discharge. These data showed that in 2001, 46% of mothers who initiated breastfeeding were continuing to mainly breastfeed at one month. A sample of 100 subjects in each group would demonstrate an increase in this "mainly breastfeeding" rate from 46% (control group) to 67% (intervention group) with alpha [confidence level] = 0.05 and power of 80%. We had assumed a more modest benefit of our PC intervention than those reported by Morrow (12% controls and 67% of a six-visit intervention group were exclusively breastfeeding at three months) and Haider (6% controls and 70% of a 15-visit intervention group were exclusively breastfeeding at five months) as these two studies had home visits as part of the intervention [7,8].

### Randomisation

**Figure 1:**
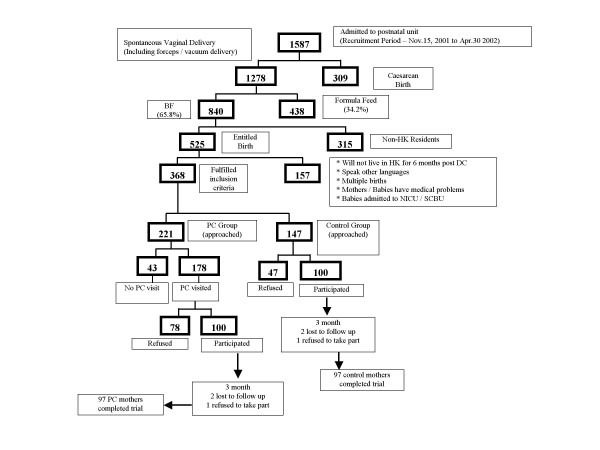
Flow of study participants from recruitment period, 15 November 2001 to 30 April 2002, to 6 months post-partum.

The hospital's PC visiting programme was conducted on an ad hoc basis at a time convenient to the PC. In view of the ad hoc visiting schedule of the PCs it was decided for recruitment purposes to allocate eligible mothers on an alternating basis, and subject to bed availability, to one of two ward areas: a PC ward area or a control ward area. The PCs were asked to only see mothers allocated to the PC ward area during the study period. Although this was done to avoid the potential influence of placing a control mother next to mother receiving PC advice, the strategy had the effect of preventing a true randomisation of mothers as enrolment occurred after allocation to these ward areas. All potentially eligible mothers were screened by one of us (Esther Wong) on the day of discharge. PC group mothers needed to have had at least one PC hospital visit and they were recruited even if they had given up breastfeeding prior to discharge to avoid over-estimating any potential benefit of the PC intervention. As the numbers of potential control group mothers exceeded the number of potential PC mothers, computer generated random number lists were used for the selection of control mothers. The recruitment process is summarised in Figure 1.

### Data collection

Recruitment data were collected through a structured interview, after obtaining signed informed consent and included information on mother's obstetric and breastfeeding history, current feeding method, preparation for breastfeeding, lactation problems, breastfeeding support network, breastfeeding duration plan, breastfeeding confidence level, breastfeeding knowledge (pre-test), information on hospital practice postpartum related to the Ten Steps of the Baby Friendly Hospital Initiative, demographic information and infant formula supplementation (if any). Each study mother was followed for 6 months after they returned home. Follow-up interviews, regardless of whether mothers had changed their feeding methods, were scheduled at five days, three months and six months post-discharge. Information collected at these interviews included current feeding practices, feeding problems, baby's health, use of pacifier, infant formula advertisement exposure, and other factors likely to influence feeding choice. The Day five and Month three interviews were telephone interviews. The Month six interview was a face-to-face interview at the mother's local MCHC at the time of a routine visit for immunisation, or was conducted by telephone for those mothers who were not able to meet at the MCHC. Evaluation of the overall breastfeeding experience, evaluation of PC support (for PC group mothers only), breastfeeding knowledge (post-test), questions on breastfeeding plans and detailed questions on infant formula advertisements were only done at the Month six interview. Mothers who stopped breastfeeding at any stage during the study completed a termination questionnaire, which explored factors for early cessation of breastfeeding. Participating PCs completed an evaluation at the end of the study to obtain feedback and comment on the PC programme. The questionnaires were based on those used in the "Perth Aboriginal Breastfeeding Study" in Australia in 2000 [9], and on information related to the "Ten Steps to Successful Breastfeeding" and the "International Code of Marketing of Breast Milk Substitutes". The questionnaires were modified after piloting.

### Recruitment of peer counsellors

The PCs were recruited from 21 mothers who participated in the hospital's first PC training course that had been jointly organised with the Baby Friendly Hospital Initiative Hong Kong Association in 2001. These mothers were required to have breastfeeding experience and to be enthusiastic to promote and support other breastfeeding mothers. The 20-hour PC course was conducted by lactation consultants and medical staff with experience in managing breastfeeding problems and involved both lectures and practical sessions on the ward. The course syllabus was based on the 40 hours WHO/UNICEF course for health professionals. After completion of the course, the PCs agreed to provide 80 hours of volunteer work in the hospital. There were 16 of these 21 trained PCs who agreed to participate in the study.

### Outcome measures and breastfeeding definitions

The primary outcome measures, breastfeeding duration and exclusivity, were compared between two groups (PC mothers vs. control mothers). As these measures were self-reported by the participating mothers, memory aids and a diary were given to mothers to reduce possible recall bias. To classify breastfeeding during the study, we used Labbok's definitions namely full breastfeeding (exclusive and almost exclusive breastfeeding), partial breastfeeding (high, medium and low) and token breastfeeding [10,11]. However a further refinement was added by including "predominant breastfeeding" practice as a category between "almost exclusive breastfeeding" and "high partial breastfeeding" categories, where "predominant breastfeeding" was defined as infrequent infant formula feeds in addition to infrequent feeds of water, juice, oral rehydration solution and ritualistic feeds. For the purposes of analysis, two different definitions of exclusive breastfeeding were used: "exclusive breastfeeding since birth" (Labbok's strict definition) which requires that no other liquid or solid (excluding vitamins, minerals and medicines) has been given to the infant since the time of birth [10,11]; and "current exclusive breastfeeding" (Aarts' definition) that describes the current exclusive breastfeeding status at the time of interview i.e. mother's feeding practice during the 24 hours period prior to the interview. [12].

### Ethics approval

The study was approved by the hospital's Ethics Committee and participating mothers were provided with written information about the study and signed informed consent.

### Statistical analysis

All data were presented as count, percentage, or mean (standard deviation, SD). Demographic characteristics, breastfeeding characteristics and background information of the infants at the baseline between the PC and control groups were compared using t-test, Mann-Whitney test, Chi-square test or Fisher exact test as appropriate. Time to terminate any breastfeeding was analysed using survival analysis. Survival (continuing to breastfeed) was estimated by Kaplan-Meier method, and the log-rank test was used to compare the overall survival rates (survival curves) between the two groups. Changes in proportions of breastfeeding practice at each of the follow-ups (Day five, Month three, Month six) relative to the baseline between the PC and control groups were assessed using mixed effects logistic regression models. Adjustment was made in the mixed effects logistic regression models for the following confounding factors: previous breastfeeding experience; primiparae or not; working status at baseline. The mixed effects models were fitted using MLwiN (version 2.02, Institute of Education, UK). All other statistical analyses were done using SPSS 11.5 (SPSS INC., Chicago, IL, USA). All statistical tests were two sided and p-value < 0.05 was considered statistically significant.

## Results

### Participant flow

Within the recruitment period, 15 November 2001 to 30 April 2002, 1587 mothers were admitted to the hospital's postnatal ward (Figure 1). Of the 221 mothers approached in the PC ward area, 178 had had a PC visit making them eligible for recruitment but 78 (44%) refused to participate. In the control ward area 147 mothers were approached and 47 (32%) refused to participate (Figure 1). Common reasons for refusal included mothers were overwhelmed by the birth of the baby, they were unsure about breastfeeding, they anticipated quitting breastfeeding right after discharge, they felt uncomfortable talking about breastfeeding, they did not need any assistance on breastfeeding (mainly in PC group), they did not like to be disturbed by phone interviews, they were in a rush to go home during the discharge period or they were not interested in participating in any research. Two mothers from each study group were lost to follow-up, and one mother from each group withdrew from the study at three months leaving a total of 194 mothers who completed the study (97 in each group) (Figure 1).

### Group differences

Compared with mothers in the control group, more primiparous mothers were recruited into the PC group (67% vs. 55%, p = 0.08), fewer mothers in the PC group had had previous breastfeeding experience (58% vs. 83%, p = 0.014), among multiparous mothers in the PC group there was a shorter mean breastfeeding duration for their first child (9.8 (± 15.8) weeks vs. 20.2 (± 23.1) weeks, p = 0.02), and more mothers in the PC group reported breastfeeding problems at discharge (89% vs. 77%, p = 0.024) (Table 1).

**Table 1:** Characteristics of the study mothers and infants participating in a study to assess the effect of a peer counselling (PC) programme on breastfeeding duration and exclusivity

	**PC group (n = 100)**	**Control group (n = 100)**
**Demographic Characteristics**		
Mean maternal age, year (SD)	29.9 (5.3)	30 (4.6)
Mean age for father, year (SD)	33.7 (7.2)	33.4 (5.8)
Mother born in Hong Kong	55%	61%
Father born in Hong Kong	69%	72%
Marital Status: married	97%	97%
Primiparae	67%	55%
Previous breastfeeding experience	57.6% * (n = 33)	82.6% * (n = 45)
Mean previous breastfeeding duration:		
1^st ^child, weeks (SD)	9.8 (15.8) *	20.2 (23.1) *
2^nd ^child, weeks (SD)	9.3 (13.6)	28 (32.3)
3^rd ^child, weeks (SD)	-	62 (27.7)
Mothers education		
Primary	4%	3%
High school	80%	89%
Tertiary	16%	8%
Fathers education		
Tertiary	20%	15%
Mothers occupation		
White collar	17%	11%
Blue collar	54%	46%
Student	0%	2%
Housewife	29%	41%
Maternal smoking status		
Non smokers	89%	96%
Ex-smokers	9%	3%
Smokers	2%	1%
Fathers smoking status		
Smokers	46%	36%
**Breastfeeding Characteristics**		
Planned pregnancy	59%	53%
When breastfeeding decision made		
Before pregnancy	30%	28%
During pregnancy	63%	63%
After the birth of the baby	7%	9%
Attended antenatal class	66%	59%
Had breastfeeding duration plan (%)	19%	28%
Report breastfeeding problem at discharge	89% *	77% *
**Background Information (infants)**		
Male gender	52%	47%
Mean gestational age, weeks (SD)	38.9 (1.2)	39 (1.4)
Mean birth weight, grams (SD)	3151 (378)	3189 (365)
Mode of delivery		
Spontaneous vaginal delivery	82%	85%
Forceps delivery	4%	7%
Vacuum delivery	14%	8%
Father present at birth	51%	41%

* Significantly different with *p *value < 0.05

### Outcomes

At discharge, there were 14% mothers in PC group and 23% of mothers in control who practised "exclusive breastfeeding since birth" (Labbok's definition) and at Day five follow-up, 46% of PC mothers and 53% of control mothers breastfed their infants exclusively or almost exclusively on breast milk (Aarts' definition) (Table 2). At Month three follow-up, only 17% of PC mothers and 23% of control mothers still exclusively or almost exclusively breastfed their infants (Aarts' definition) and 66% of PC mothers and 59% of control mothers had given up breastfeeding or were lost to follow-up (Tables 2 and 3). At the Month six follow-up, 18% of PC mothers and 18% of control mothers were continuing to breastfed their infants exclusively or almost exclusively (Aart's definition). There was no significant difference in these rates at any time point (Table 2). Reasons for giving up breastfeeding at the different time points included low milk supply, mother returning to work, maternal fatigue and mother found breastfeeding difficult to do (Table 4).

**Table 2:** Breastfeeding practices across time showing unadjusted Odds Ratios (95%CI)

	**PC group (n = 100)**	**Control group (n = 100)**	**Unadjusted Odds Ratio^1^(95% CI)**	**p-value**
**Exclusive breastfeeding**				
Baseline^2^	14 (14%)	23 (23%)	0.55 (0.26, 1.13)	0.24^4^
Day 5^3^	37 (37%)	38 (38%)	0.96 (0.54, 1.70)	0.23^5^
Month 3^3^	10 (10%)	9 (9%)	1.12 (0.44, 2.90)	0.20^5^
Month 6^3^	2 (2%)	1 (1%)	2.02 (0.18, 22.7)	0.35^5^
				
**Exclusive OR almost exclusive breastfeeding**				
Baseline^2^	14 (14%)	23 (23%)	0.55 (0.26, 1.13)	0.49^4^
Day 5^3^	46 (46%)	53 (53%)	0.76 (0.43, 1.32)	0.64^5^
Month 3^3^	16 (17%)	22 (23%)	0.67 (0.33, 1.38)	0.68^5^
Month 6^3^	17 (18%)	17 (18%)	1.00 (0.48, 2.10)	0.25^5^
				
**Exclusive, almost exclusive OR predominant breastfeeding**				
Baseline^3^	60 (60%)	71 (71%)	0.61 (0.34, 1.10)	0.32^4^
Day 5^3^	57 (57%)	63 (63%)	0.78 (0.44, 1.37)	0.46^5^
Month 3^3^	19 (20%)	25 (26%)	0.70 (0.36, 1.38)	0.50^5^
Month 6^3^	17 (18%)	18 (19%)	0.93 (0.45, 1.94)	0.22^5^

^1^Odds Ratio of breastfeeding practice (PC group vs Control group) at each time point.

^2^Exclusive breastfeeding since birth (Labbok's strict definition).

^3^Exclusive breastfeeding current status (Aarts' definition).

^4^p-value testing the difference in baseline breastfeeding practice between the PC and control groups after adjusting for having or not previous breastfeeding experience, primiparae or not, and working status (having or not worked in the last 12 months).

^5 ^p-value testing the difference in change from baseline in breastfeeding practice between the PC and control groups after adjusting for having or not pervious breastfeeding experience, primiparae or not and working status (having or not worked in the last 12 months).

**Table 3:** Survival table for breastfeeding duration among 200 mothers participating in a study to assess the effectiveness of a peer counselling (PC) programme

	**Day 5**		**Month 3**		**Month 6**	
**Study groups**	**PC**	**Control**	**PC**	**Control**	**PC**	**Control**

Cumulative % of mothers breastfeeding	87%	88%	36%	40%	24%	31%
Mothers ceased breastfeeding (n)	13	12	63	57	74	66
Lost to follow-up/drop-out	0	0	3	3	3	3

**Table 4:** The most common reasons# given by mothers for stopping breastfeeding

	**Peer counsellor group**	**Control group**
**Before the first follow-up (Day 5)**	n = 13	n = 12
Breastfeeding was tough and difficult work	9 (69%)	9 (75%)
Maternal fatigue	9 (69%)	8 (67%)
Attachment and feeding technique promblems	8 (62%)	4 (33%)
Low milk supply	7 (54%)	7 (58%)
Anxious/unsure about breastfeeding	6 (46%)	2 (17%)
		
**From day 5 post discharge to month 3 follow-up**	n = 50	n = 45
Returned to work	26 (52%)	21 (47%)
Maternal fatigue	22 (44%)	21 (47%)
Low milk supply	19 (38%)	16 (36%)
Breastfeeding was tough and difficult work	14 (28%)	12 (27%)
Too much motivation was required	13 (26%)	14 (31%)
		
**Between month 3 and month 6 follow-up**	n = 11	n = 9
Low milk supply	3 (27%)	5 (56%)
Baby was old enough not to be breastfed	3 (27%)	1 (11%)
Mother had already given a good start	3 (27%)	1 (11%)
Baby bites nipples	2 (18%)	2 (22%)
Maternal fatigue	2 (18%)	1 (11%)

^# ^Multiple reasons were given by some mothers.

### Ancillary analyses

**Figure 2:**
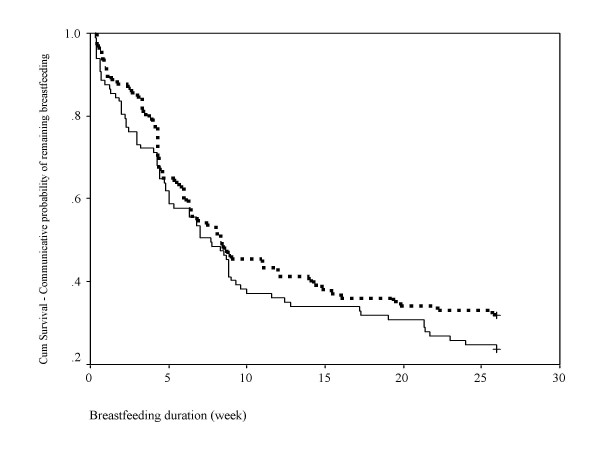
Kaplan-Meier survival plots of the breastfeeding duration for the intervention (n = 100, solid line) and control (n = 100, broken line) groups.

Kaplan-Meier survival plots of the breastfeeding duration for the two study groups (Figure 2) were not significantly different, log-rank test, p = 0.24. The survival rates of practising any breastfeeding at Month six were 24% with median survival time of 7.7 weeks (95% confidence interval (CI): 6.0, 9.4 weeks) for the PC group and 32% with median survival time of 8.3 weeks (95% CI: 4.6, 12 weeks) for the control group. The proportion of mothers continuing to breastfeed at Day five, Month three and Month six were 87% PC vs 88% Control (p = 0.78), 36% PC vs 41% Control (p = 0.35), and 24% PC vs 32% Control (p = 0.24), respectively (Table 3). None of the 97 PC mothers reported that the peer support was too frequent, over half felt that the overall frequency of the PC support was just adequate, and a few mothers reported that the support was inadequate to help them solve their breastfeeding problems.

### Process evaluation

To evaluate the PCs' assessment of the programme, evaluation questionnaires were distributed within two weeks of the trial completion and returned by 11 of the 16 PCs. Telephone consultation time ranged from one minute to 80 minutes. There were three commonly discussed topics related to breastfeeding: insufficient breast milk (82%), frequent feeding (46%), and breastfeeding technique (36%). The main topics related to babies were sleepy baby (46%), information on general baby care (36%) and baby's stool pattern (36%). Key topics related to mothers were breast engorgement and mastitis (46%), emotional problems and handling techniques (18%) and maternal fatigue (18%). Using 10-point rating scales, PCs evaluated the efficiency of the PC program (10 = strongly agree; 1 = strongly disagree). The mean score (± SD) of the overall effectiveness of the program was 8 ± 1.3 (range 6 – 10), with the hospital visits being 8.8 ± 1.2 (range 7 – 10) and the telephone follow-up being 8.2 ± 1.9 (range 4 – 10). The mean score (± SD) for the adequacy of the numbers of scheduled telephone contacts for new breastfeeding mothers was 4.4 ± 3.2 (range 1 – 10). Four PCs rated their confidence at 9, three at 8, three at 7 and one at 5. The mean score (± SD) of the PCs level of enjoyment in supporting breastfeeding mothers through hospital visit and telephone consultations was 7.9 ± 1.4 (range 6 – 10) and 8.1 ± 1.4 (range 6 – 10) respectively. The aspects which participating PCs liked the most were the sense of being supportive (91%) of breastfeeding mothers and their babies, and a sense of satisfaction (73%). In contrast, the aspects which PCs disliked the most were being rejected when offering assistance (36%) and the strong misconceptions about breastfeeding (18%) by some mothers. Although, PCs were encouraged to give their phone numbers to study mothers for two-way contact, only three out of sixteen PCs did so. Although PCs acknowledged that "two-way contact" was likely to be of greatest benefit, most responded that the "one-way contact" model worked best for them in view of time constraints and other commitments. Some PCs noted that it was difficult to keep to the fixed schedule as mothers could not always be contacted. Three PCs responded that they would definitely take part in the program again if it was repeated, seven PCs reported that they probably would do so, and one PC said that she was not sure.

## Discussion

There was no significant difference in the overall breastfeeding duration or the exclusivity of breastfeeding between mothers who received routine breastfeeding support and advice (control group) and mothers who received additional PC support consisting of a hospital visit and follow-up telephone support (PC group). These results contrast with many studies undertaken elsewhere [5,7,8,13,14] We considered three possible explanations for our findings. First, the result could have been due to local and cultural factors that result in a low baseline rate of breastfeeding, particularly exclusive breastfeeding, in Hong Kong. Second, the result could have been due to the nature of the PC intervention used and third, the result could have been due to the group selection process.

Local perceptions of the value of breastfeeding could have prevented an effective PC programme from increasing breastfeeding duration or exclusivity. Any intervention alone may not be strong enough to overcome powerful prevailing cultural norms that favour infant formula feeding. One study that did not show any impact of a PC intervention was undertaken in Glasgow, Scotland [15]. Hong Kong, like Glasgow, is a city where breastfeeding rates are low, where breastfeeding appears not to be highly valued by the community and where infant formula feeding is portrayed as the publicly recognised social norm. In an international study of child care practices, Scotland like Hong Kong showed low rates of breastfeeding only (26%) and high rates of infant formula feeding only (58%) at three months of age [4]. In contrast, Dennis' trial which showed a beneficial effect of a telephone-based peer support programme was conducted in a community with high breastfeeding rates [5]. A low acceptability of breastfeeding in society may impact adversely on breastfeeding success [16-18], and could reduce the benefit of a potentially effective PC programme.

The second explanation for our results could be that the PC intervention itself was ineffective. Our PC programme provided at least one hospital visit and a total of seven telephone follow-up contacts at fixed time intervals after the mothers had returned home. Although the number of contacts were generally considered adequate by mothers, it was not always possible for PCs to stick to the schedule. Also such fixed schedules may not suit the individual mother's need for lactation support. Our programme was effectively a "one-way contact" in which PCs mainly initiated phone calls to breastfeeding mothers.

Some PC interventions have started postpartum, whereas other programmes have arranged for the PC to meet the pregnant women during the antenatal period before the birth experience [7,8,13-15,19] Although we are aware of no study comparing interventions started in the antepartum period with those started only in postpartum period, it is likely that the longer the period that a mother is assigned to a PC, the higher probability that the PC will be included as a member of the mother's support network, and the more influence the PC will have on the mother's breastfeeding decision and later breastfeeding practice))[20]. It has been suggested that qualitative research should explore the different elements of breastfeeding support strategies and the mechanisms by which support operates [6].

Only mothers with vaginal births were recruited in our study. It is possible that our PC programme could have been beneficial to mothers experiencing a caesarean delivery who may have more problems initiating breastfeeding and who may need more support. Although our PC programme did not appear to prolong breastfeeding duration or increase breastfeeding exclusivity, it did improve the breastfeeding knowledge of those mothers who received the PC intervention (data not shown). The PCs also reported positive growth in their own personal life, in terms of knowledge of breastfeeding and a sense of achievement and self-confidence.

Subjects were recruited prior to discharge after they had been allocated to different ward areas and after the PC group had received at least one PC visit. This recruitment strategy resulted in important differences between the PC and control groups (Table 1). Although we controlled for these differences in the analysis, it is possible that there were additional factors, not controlled for, that could explain why our PC programme did not appear to be effective. Our study highlights the importance of carefully considering the randomisation process when conducting trials that assess complex interventions [21]. Future studies evaluating PC programmes will need to carefully consider the method of randomisation as this aspect has also proved to be problematic in some other studies (Table 5). These variations in recruitment strategies highlight some of the difficulties of conducting such studies. The Medical Research Council has suggested a framework for the design and evaluation of complex interventions which can include exploratory phase II studies [21]. Such a strategy could have identified limitations of our design.

**Table 5:** Comparison of randomisation methods and intervention of studies evaluating peer counseling programmes

**Study**	**Randomisation**	**Sample (n)**	**Intervention**	**Outcomes**
Kistin et al 1994 [13]	Compared women who planned to breastfeed and received support from counsellors (n = 59) with those who requested counsellors but, owing to inadequate counsellors, did not have a counsellor (n = 43).	102	Trained counsellors matched by race if possible to low-income pregnant or postpartum women. Contact prior to delivery encouraged and then telephone contact every 1 to 2 weeks until two months and then "as needed".	Breastfeeding initiation, exclusivity and duration.
Schafer et al 1998 [14]	2 "intervention counties" and 6 "control counties". All women referred to Women, Infants and Children's Nutrition programme centres in these counties were recruited.	207	The assignment of trained volunteers with previous successful personal experience with breastfeeding as peer counsellors to low-income pregnant women. Peer counselor met mother antenatally. Maintained telephone contact between visits.	Initiation and duration of breastfeeding
Arlotti et al 1998 [19]	Convenience sample from prenatal and postpartum clients who were assigned to counsellors based on their desire to have a counsellor and availability of counsellors. Mothers who were not matched with a counsellor were the control group.	36	Counsellors contacted mothers within a few days of delivery and again at 2 w, 1 m, 2 m and 3 m. Further contacts by telephone, letter or in person at clinic	Exclusive breastfeeding at 2 weeks, 1 month, 2 months and 3 months and duration of exclusive breastfeeding
Morrow et al 1999 [8]	Cluster randomisation before the recruitment of study mothers. City blocks were the unit of randomisation.	130	3 or 6 counselling home visits antenatally and early postpartum	Exclusive breastfeeding at 3 months and duration of breastfeeding
Haider et al. 2000 [7]	Cluster randomisation. city area divided into zones and random number tables used to randomly select zones into 2 study groups Thereafter, mothers were approached in the community and invited to participate.	726	15 home-based counselling visits scheduled with two visits in last trimester, three early postpartum and then every two weeks til 5 months	Exclusive breastfeeding at 5 months
McInnes at al 2000 [15].	Mothers recruited antenatally by a clerical officer in two culturally and socially similar but geographically separated communities in Glasgow.	995	An offer to the intervention group of having visits from a "helper" with a minimum of 4 visits (2 antenatally and 2 postnatally), irrespective of breastfeeding intention.	Breastfeeding at 6 weeks
Dennis et al. 2002 [5]	Randomised eligible mothers after they had been recruited and had signed written informed consent. Used randomly generated numbers and sequentially numbered sealed opaque envelopes. Mothers recruited from 2 semi-urban community hospitals near Toronto.	256	Telephone-based support, within 48 hrs of hospital discharge	Breastfeeding (any or exclusive) at 1,2, and 3 months

In addition it is possible that even with good randomisation in a community setting, an effective peer counselling intervention could influence practice within the wider community in highly urbanised settings such as Hong Kong i.e. it may not be possible to prevent the control mothers from getting access to the intervention. Although a systemic review has shown lay support to be effective in reducing the cessation of exclusive breastfeeding, it is suggested that further trials are required to assess the effectiveness of this support in different settings, particular in those communities with low rates of breastfeeding initiation [6].

## Conclusion

A mainly telephone contact PC programme did not increase breastfeeding duration and exclusivity in our setting. It is possible that the somewhat limited PC intervention programme that we used and a low value placed on breastfeeding in Hong Kong society could help explain this result. Group differences could have also been a factor although these were controlled for in the analysis. It is possible that effective PC programmes in areas with prevailing "infant formula feeding" cultures may have limited impact unless these are part of more extensive programmes to implement all of the "Ten Steps" in entirety and other community measures to increase the cultural acceptability of breastfeeding. In future, consideration should be given to evaluating or implementing alternative PC programmes, such as those that link a PC to an individual mother antenatally.

## Abbreviations

PC - Peer counsellor;

WHO - World Health Organization;

UNICEF - United Nations Children's Fund;

MCHC - Maternal and Child Health centre.

## Competing interests

The author(s) declare that they have no competing interests.

## Authors' contributions

EHYW participated in design, acquisition, analysis and interpretation of data, drafting the manuscript and final approval of version to be published. EASN participated in design, interpretation of data, drafting the manuscript and final approval of version to be published. KCC participated in analysis and interpretation of data, drafting the manuscript and final approval of version to be published. KPW participated in design, help with acquisition of data, critical review of manuscript and final approval of version to be published. CI participated in design, help with acquisition of data, critical review of manuscript and final approval of version to be published. LCH participated in design, critical review of manuscript and final approval of version to be published.

## Funding

EHY Wong was supported by a research studentship from the Research Grants Council, Hong Kong. The funding body had no role in the study design; collection, analysis and interpretation of data; writing the manuscript; or decision to submit the manuscript for publication.
